# Supplementary material for: Splice-Junction-Based Mapping of Alternative Isoforms in the Human Proteome
Source: Cell Rep. Author manuscript; Available in PMC 2020 Jan 15. (PMC6961840; doi:10.1016/j.celrep.2019.11.026)
Supplement: 2 [file NIHMS1546469-supplement-2.zip › DF1/PXD009737/TestisTrypsin/TestisTrypsin_55_HERC4_ITIEQVPDFQGLIGQLGLGGIDEEIVLEPR.pdf]

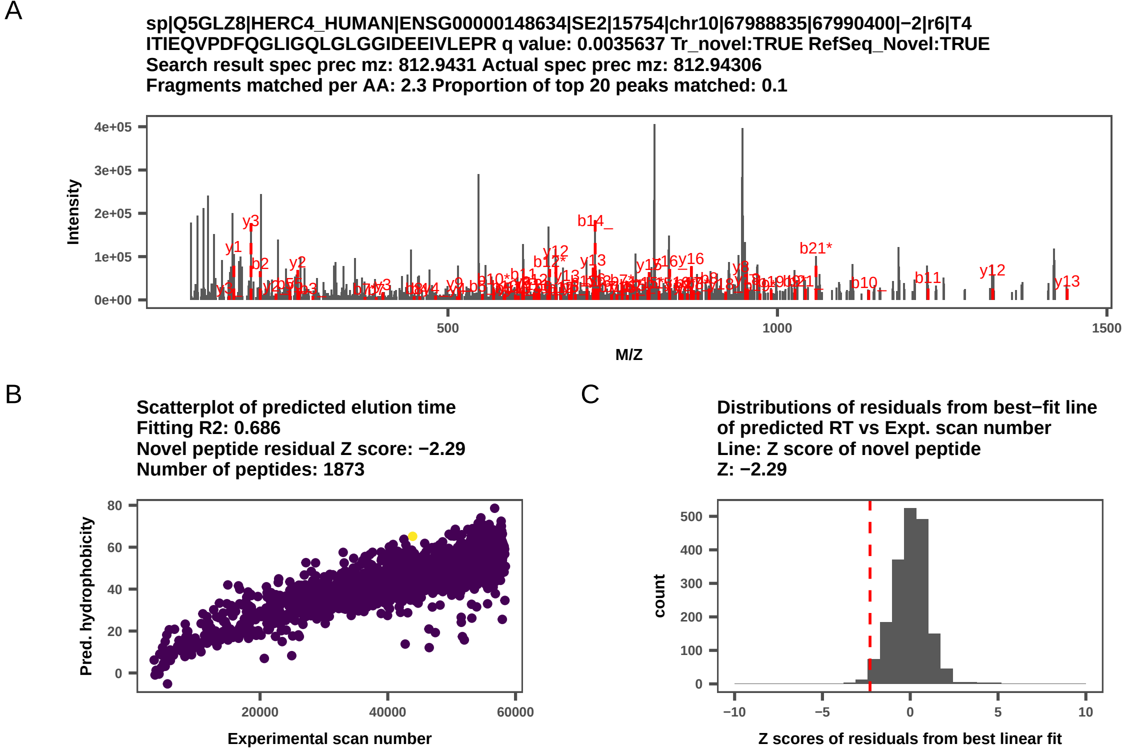
A
sp|Q5GLZ8|HERC4_HUMAN|ENSG00000148634|SE2|15754|chr10|67988835|67990400|−2|r6|T4
ITIEQVPDFQGLIGQLGLGGIDEEIVLEPR q value: 0.0035637 Tr_novel:TRUE RefSeq_Novel:TRUE
Search result spec prec mz: 812.9431 Actual spec prec mz: 812.94306
Fragments matched per AA: 2.3 Proportion of top 20 peaks matched: 0.1
Intensity
4e+05
3e+05
2e+05
1e+05
0e+00
y3
y1
b2
y2
b14_
b21*
y12
b12*
y13
y16
y8
b10_
b11
y12
y13
M/Z
500
1000
1500
B
Scatterplot of predicted elution time
Fitting R2: 0.686
Novel peptide residual Z score: −2.29
Number of peptides: 1873
Pred. hydrophobicity
80
60
40
20
0
20000
40000
60000
Experimental scan number
C
Distributions of residuals from best−fit line
of predicted RT vs Expt. scan number
Line: Z score of novel peptide
Z: −2.29
count
500
400
300
200
100
0
−10
−5
0
5
10
Z scores of residuals from best linear fit
